# Validation of Calprotectin As a Novel Biomarker For The Diagnosis of Pleural Effusion: a Multicentre Trial

**DOI:** 10.1038/s41598-020-62388-y

**Published:** 2020-03-30

**Authors:** Maribel Botana-Rial, Lorena Vázquez-Iglesias, Pedro Casado-Rey, María Páez de la Cadena, María Amalia Andrade-Olivié, José Abal-Arca, Laura García-Nimo, Lucía Ferreiro-Fernández, Luis Valdés-Cuadrado, María Esther San-José, Francisco Javier Rodríguez-Berrocal, Alberto Fernández-Villar

**Affiliations:** 1 Pulmonary Department, Hospital Álvaro Cunqueiro, EOXI Vigo, PneumoVigoI+I Research Group, Health Research Institute Galicia Sur (IIS Galicia Sur), Vigo, Spain; 2 Department of Biochemistry, Genetics and Immunology, Faculty of Biology, University of Vigo, Vigo, Spain; 3 Clinical Chemistry Department, Hospital Álvaro Cunqueiro, EOXI Vigo, Vigo, Spain; 4 Pulmonary Department, University Hospital Complex in Ourense, EOXI Ourense, Ourense, Spain; 5 Clinical Chemistry Department, University Hospital Complex in Ourense, EOXI Ourense, Ourense, Spain; 6 Pulmonary Department, University Hospital Complex in Santiago, EOXI Santiago, Health Research Institute Santiago (IDIS), Santiago de Compostela, Spain; 7 Clinical Chemistry Department, University Hospital Complex in Santiago, EOXI Santiago, Santiago de Compostela, Spain

**Keywords:** Tumour biomarkers, Diagnostic markers

## Abstract

Discriminating between malignant pleural effusion (MPE) and benign pleural effusion (BPE) remains difficult. Thus, novel and efficient biomarkers are required for the diagnosis of pleural effusion (PE). The aim of this study was to validate calprotectin as a diagnostic biomarker of PE in clinical settings. A total of 425 patients were recruited, and the pleural fluid samples collected had BPE in 223 cases (53.7%) or MPE in 137 patients (33%). The samples were all analysed following the same previously validated clinical laboratory protocols and methodology. Calprotectin levels ranged from 772.48 to 3,163.8 ng/mL (median: 1,939 ng/mL) in MPE, and 3,216–24,000 ng/mL in BPE (median: 9,209 ng/mL; *p* < 0.01), with an area under the curve of 0.848 [95% CI: 0.810–0.886]. For a cut-off value of ≤ 6,233.2 ng/mL, we found 96% sensitivity and 60% specificity, with a negative and positive predictive value, and negative and positive likelihood ratios of 96%, 57%, 0.06, and 2.4, respectively. Multivariate analysis showed that low calprotectin levels was a better discriminator of PE than any other variable [OR 28.76 (*p* < 0.0001)]. Our results confirm that calprotectin is a new and useful diagnostic biomarker in patients with PE of uncertain aetiology which has potential applications in clinical practice because it may be a good complement to cytological methods.

## Introduction

The diagnosis of pleural effusion (PE) is a clinical challenge because it can be produced by over 60 diseases^1,2^. Nevertheless, in clinical practice the priority is to establish whether the PE is malignant or not. A diagnosis of malignant PE (MPE) implies the presence of advanced-stage tumours and is therefore associated with a poor prognosis^1,3^ which requires urgent diagnosis.

Thoracocentesis is the first and most simple procedure for the diagnosis of PE^2–4^. Unfortunately, while its specificity for establishing malignancy is 100%, the diagnostic sensitivity of pleural fluid (PF) cytological analysis is low. Although the odds of establishing an MPE diagnosis by immunohistochemistry are improved by applying a panel of different antibodies, its diagnostic sensitivity is still only approximately 60% for metastatic PE and less than 30% for mesothelioma^5,6^.

When the cytology results are negative, more invasive methods such as a pleural biopsy or thoracoscopy are necessary^2,4,7^. In this context, more groups are searching for PF biomarkers for malignancy with the aim of avoiding these invasive procedures^8–10^. Recent meta-analyses have evaluated the ability of new biomarkers such as survivin, vascular endothelial growth factor, fibulina-3, or combinations of tumour markers^11–14^ to improve the diagnosis of PE. However, the results achieved so far are inconclusive and novel non-invasive biomarkers for the precise diagnosis of malignancy in PE are still required.

In two previous studies, one conducted in a single centre and the other in a research laboratory^15,16^, we concluded that measuring calprotectin levels in PE could predict malignancy with a high degree of accuracy. The limitations of these studies were that calprotectin was measured using a non-automated commercial kit and the patients came from a single hospital^16^. Thus, the aim of this multicentre prospective study was to validate calprotectin levels as a diagnostic biomarker of MPE in patients from three different hospitals during routine clinical practice.

## Methods

### Study design and patient selection

This study was conducted at three centres in Spain: the University Hospital Complex in Vigo (coordinating centre; Centre 1), the University Hospital Complex in Santiago de Compostela (Centre 2), and the University Hospital Complex in Ourense (Centre 3). Patients with PE admitted in the participating hospitals from January 2014 to June 2016 were consecutively enrolled into this study. The same protocol was used at all three participating centres. The inclusion criteria were: (1) patients aged over 18 years; (2) presence of transudate or exudate PE with a specific diagnosis; (3) patient provided their signed written and informed consent to participation. The exclusion criteria were: (1) previous pleurodesis diagnosis; (2) current treatment with intrapleural or systemic anti-neoplastic agents; (3) the presence of pus in the pleural space (empyema); or (4) PE of an unknown aetiology (when the procedure for PE diagnosis was not completed and its cause was uncertain).

The sample size was calculated using the results obtained in our previous study^14^. Considering that calprotectin determination had a sensitivity of 97% and a specificity of 89% (for a cut-off value of ≤ 545 ng/mL), and assuming 20–40% were MPEs, with a probability of sensitivity accuracy estimation error of 3%, the required sample size was 394 patients.

### Pleural effusion diagnosis

The PE diagnosis protocol followed the recommendations set out in the guidelines published by different medical societies^2,4^ and was employed in previous studies by our group^15,16^. The patients underwent a baseline assessment, which included taking their medical history, completing a complete physical examination, and undertaking a chest radiography. In addition, a diagnostic thoracocentesis for biochemical, microbiological, and cytological studies was performed in all the patients. If a diagnosis was not obtained after this procedure, an ultrasound-guided pleural needle-biopsy was performed in individuals with exudative PE.

Subsequently, and at the pulmonologist’s discretion, patients in whom the cause of PE had not yet been identified were submitted for medical or surgical thoracoscopy or were followed-up with clinical and radiological examinations for at least 1 year to confirm the resolution of their symptoms and confirm that PE had not recurred. Light’s criteria were used to differentiate between PF exudates and transudates^17^. The aetiology of PE was determined based on accepted criteria, as described by different medical societies^2,4^. Patients with exudates were classified into five main groups according to the cause of the PE as follows.

MPE was defined by the presence of malignant cells in PF cytology or pleural biopsy. Tuberculous PE was defined by the presence of pleural caseating granulomas; a lymphocytic-predominant exudate and high adenosine deaminase (ADA) levels inasuitable non-clinical epidemiological context; or after detection of *Mycobacterium tuberculosis* in patient sputum, pleural fluid (PF), or pleural biopsy specimens (either by microscopy or cell culture).

Parapneumonic PE was defined as PE associated with bacterial pneumonia, a lung abscess, or bronchiectasis.

Non-malignant PE was defined as nonspecific pleuritis observed during thoracoscopy, thoracotomy, or autopsy (idiopathic PE); or the absence of symptoms or non-recurrence of PE during a 1-year follow-up period (reactive PE).

Miscellaneous PE was diagnosed based on predefined criteria from medical guidelines and defined patients who had not been included in any of the previous groups.

### Demographic, clinical, radiological, and biochemical variables

Demographic data such as gender, smoking status, previous cancer diagnosis, age, and other clinical variables including chest pain, dyspnoea, cough, fever, and weight loss were recorded. The radiological size of the PE in the chest radiographs was classified according to the following criteria: as ‘PE occupying more than two thirds of the chest’ when the PE produced opacification of the entire hemithorax or when the fluid reached the arch of the aorta, and otherwise as ‘PE occupying less than two thirds of the chest’. The PF biochemical parameters, differential cell counts, pH, proteins, levels of lactate dehydrogenase (LDH), glucose, and ADA, were determined in the Department of Clinical Chemistry at each participating hospital using automated assays according to the manufacturer’s instructions.

### Collection and processing of pleural fluid samples

PF samples were taken by thoracocentesis or during pleural biopsy before starting any treatment. Five mL of PF was obtained and introduced into a pipe with or without an anticoagulant, depending on the standard processing protocol used at each centre. The PF was then centrifuged at 800 g for 15 minutes and was immediately frozen in 0.5 mL aliquots at −80 °C. All the samples were included in the CHUVI Biobank (RETIC-FIS-ISCIII RD09/0076/00011).

### Calprotectin determination

The samples were analysed in each participating centre following a common protocol and independently of the clinical diagnosis. Calprotectin levels were determined using a sandwich ELISA (fCALTM-EK-CAL) from Bühlmann Laboratories AG, according to the manufacturer’s guidelines, with some variations to adapt it to the determination of calprotectin in PF. Samples were diluted 1/100. This methodology was previously described and evaluated to confirm its validity and robustness^18^. The assay was performed with the DSXTM Automated System (DYNEX Technologies, Worthing, West Sussex-UK) according to the manufacturer’s instructions. The absorbance was read at 450 nm and calprotectin concentrations were calculated using the known concentration calibrators provided in the kit and a 4-parameter logistic regression model (4PL).

### Ethical approval and consent to participate

Patient data and PF samples were obtained in full compliance with the clinical and ethical practices of the Spanish Government and the Helsinki Declaration. The study protocol was approved by the Galicia Ethics Committee (2014/053). All the patients received written and oral information prior to their inclusion in the study and provided written informed consent before its commencement. Participant anonymity was maintained in all cases.

### Statistical methods

Kolmogorov–Smirnov and Levine tests were applied to continuous variables to verify their normal distribution and homogeneity of their variances, respectively. When the normal distribution hypothesis was rejected, we conducted our analyses with non-parametric tests: Mann–Whitney U tests were used to evaluate the differences between independent samples and Kruskal–Wallis tests for multiple comparisons. The results are given as the median (25th–75th percentiles) for quantitative variables and as percentages and absolute frequencies for qualitative variables.

The accuracy of calprotectin levels to discriminate MPE from BPE was evaluated using receiver operating characteristic (ROC) curves. Univariate logistic regression was performed to test the association between calprotectin levels and the presence of MPE; this relationship was also examined for other variables. The unadjusted odds ratio (OR), calculated as an estimate of the relative risk, and the corresponding 95% confidence intervals (CI) were reported. Significant predictors in the univariate analysis (*p* < 0.1) were entered into a multivariate logistic regression model to assess the independent predictive value of calprotectin levels, considering a *p*-value of <0.05 as statistically significant. The data were analysed using SPSS statistical software (version 21, IBM Corp., Armonk, NY).

## Results

This study included 415 consecutive cases of PE referred to the three participating hospitals; 223 (53.7%) were BPE, 137 (33%) were MPE, and there were 55 (13.3%) transudates. The aetiologies of these PEs are shown in Table 1. Out of the 313 (75.4%) cases of PE with negative cytology results, 35 (11.2%) were MPEs; 112 (27%) patients underwent closed pleural biopsy: 57 (50.9%) were BPEs, 52 (46.4%) MPEs, and 3 (2.7%) transudates.

**Table 1:** Aetiology of pleural effusion in the study population, by centre.

Causes of pleural effusion	All patients *n* (%)	Centre 1	Centre 2	Centre 3
Benign pleural effusion	223 (53.7%)	119 (58.3%)	54 (49.1%)	50 (49.5%)
Tuberculous PE	27 (12.1%)	15 (12.6%	4 (7.4%)	8 (16%)
Parapneumonic PE	88 (39.5%)	48 (40.3%)	29 (53.7%)	11 (22%)
Non-malignant PE	49 (22%)	33 (27.7%)	8 (14.8%)	8 (16%)
Miscellaneous PE	59 (26.5%)	23 (19.3%)	13 (24.1%)	23 (46%)
Malignant pleural effusion	137 (33%)	56 (27.5%)	44 (40%)	37 (36.6%)
Non-small cell lung cancer	12 (49.7%)	6 (10.7%)	3 (6.8%)	3 (8.1%))
Adenocarcinoma	56 (40.9%)	24 (42.9%)	18 (40.9%)	14 (37.8%)
Small-cell lung cancer	8 (5.8%)	4 (7.1%)	3 (6.8%)	1 (2.7%)
Ovarian cancer	12 (8.7%)	6 (10.7%)	4 (9.1%)	2 (5.4%)
Gastric cancer	5 (3.6%)	2 (3.6%)	1 (2.3%)	2 (5.4%)
Breast cancer	9 (6.6%)	6 (10.7%)	1 (2.3%)	2 (5.4%)
Unknown origin	4 (2.9%)	1 (1.8%)	—	3 (8.1%)
Haematological cancers	11 (8%)	1 (1.8%)	8 (18.2%)	2 (5.4%)
Mesothelioma	11 (8%)	4 (7.1%)	4 (9.1%)	3 (8.1%)
Others*	9 (6.5%)	2 (3.6%)	2 (4.5%)	5 (13.5%)
Transudate	55 (13.3%)	29 (14.2%)	12 (10.9%)	14 (13.9%)
Heart failure	44 (80%)	23 (79.3%)	11 (91.7%)	10 (76.9%)
Hepatic hydrothorax	6 (10.9%)	4 (13.8%)	1 (8.3%)	1 (7.7%)
Nephrotic syndrome or dialysis	1 (1.8%)	—	—	1 (7.7%)
Others**	4 (7.3%)	2 (6.9%)	—	2 (14.2%)

Abbreviations: PE = pleural effusion.

*2 melanoma, 2 urological cancer, 1 metastatic soft-tissue sarcoma, 1 oesophagus carcinoma, 1 hepatocellular carcinoma, 1 kidney carcinoma, 1 colon adenocarcinoma.

**2 pericarditis, 1 amyloidosis, 1 non-specific.

Thoracoscopy was performed in 30 (7.2%) cases and 17 (56.6%) of these patients were diagnosed as having MPE. The other 13 cases (43.3%) were diagnosed with non-specific fibrinous pleuritis. The demographic characteristics and symptoms of the study population are shown in Table 2 for the 415 patients included in the study, and these results are broken down by centre in Supplementary Table S1.

**Table 2:** Demographic characteristics and symptoms of the study population.

Variable	BPE	MPE	TRANSUDATE
Gender (male/female)	145/78	76/61	41/14
Age (years)^a^	65 (50.7–77.2)	73 (61.2–83)	78 (69–85)
Tobacco	108 (48.4%)	74 (54%)	27 (49.1%)
Cancer	39 (17.5%)	42 (30.7%)	13 (23.6%)
Dyspnoea	141 (63.2%)	114 (83.2%)	48 (87.3%)
Pain chest	103 (46.2%)	46 (33.6%)	9 (16.4%)
Weight loss	15 (6.7%)	34 (24.8)	2 (3.6%)
Fever	72 (32.3%)	7 (5.1%)	4 (7.3%)
Cough	82 (36.8%)	39 (28.5%)	14 (25.5%)
Radiological size^b^	24 (10.8%)	44 (32.1%)	7 (12.7%)

Abbreviations: BPE = benign pleural effusion, MPE = malign pleural effusion.

Data are presented as a absolute frequencies and percentage.

^a^Data are presented as the median (25th–75th percentiles) (25th–75th percentiles).

^b^Pleural effusion size in the chest radiographs: PE occupying more than two thirds of the chest’ when the PE produced opacification of the entire hemithorax or when the fluid reached the arch of the aorta.

The biochemical parameters of these PE cases are shown in Table 3 and are separated by centre in Supplementary Table S2. The patient symptoms and biochemical parameters of PE were similar in all three participating centres, thus indicating a high level of homogeneity in the study population. The calprotectin concentrations in the different diagnostic groups are presented in Table 4; the calprotectin levels in MPE ranged from 772.48 to 3,163.8 ng/mL (median: 1,939 ng/mL) and were clearly lower than in the BPE samples (3,216 to 24,000 ng/mL; median: 9,209 ng/mL). In transudates, the calprotectin levels ranged from 400.0 to 548.5 ng/mL (median: 400.0 ng/mL).

**Table 3:** Pleural fluid biochemical parameters.

Variable	BPE	MPE	TRASUDATE
ADA (U/L)	24 (18.5–39.4)	20 (15–24.6)	13.9 (11–17)
LDH (U/L)	499.5 (313.5–918.5)	594 (345–887.5)	163 (146–224)
Protein (g/dL)	4.4 (3.6–4.9)	4.1 (3.3–4.7)	2.4 (1.9–3.2)
pH	7.4 (7.33–7.45)	7.4 (7.3–7.5)	7.47 (7.43–7.5)
Glucose (mg/dL)	93 (71–11)	102.5 (77–126)	113 (94–137)
Lymphocytes (%)	70 (36–92)	82 (55–93)	93 (77.5–97)
Neutrophils (%)	23 (5–60)	10 (4.7–23.2)	6 (3–20.5)

Abbreviations: BPE = benign pleural effusion, MPE = malign pleural effusion, ADA = adenosine deaminase, LDH = lactate dehydrogenase.

Data are presented as the median (25th–75th percentiles).

**Table 4:** Calprotectin concentrations in pleural fluid (ng/mL).

Causes of pleural effusion	*n* (%)	Calprotectin ngmL^−1^ median and range	*p*-value
Benign pleural effusion	223	9209 (3216–24000)	<0.001^a^
Tuberculous PE	27 (12.1%)	24000 (6850–24000)	
Parapneumonic PE	88 (39.5%)	24000 (7481–24000)	
Non-malignant PE	49 (22%)	3216 (1718–11597)	
Miscellaneous PE	59 (26.5%)	5202 (3142.8–16304)	
Malignant pleural effusion	137	1939 (772.48–3163.8)	<0.001^b^
Non-small cell lung cancer	68 (49.7%)	2895 (849–4735)	
Adenocarcinoma	56 (40.9%)	2172.5 (1184.9–3170.1)	
Small-cell lung cancer	8 (5.8%)	1686 (508.7–2916.2)	
Ovarian cancer	12 (8.7%)	2092.5 (689–2096.9)	
Gastric cancer	5 (3.6%)	1351.8 (751–3186.3)	
Breast cancer	9 (6.6%)	1118 (442.7–2639)	
Unknown origin	4 (2.9%)	1160 (506.5–4010.5)	
Hematologic cancers	11 (8%)	1570 (400–2953)	
Mesothelioma	11 (8%)	1118 (400–5186.9)	
Other*	9 (6.5%)	2952 (1470–4498.5)	
Transudate	55	400 (400–548.5)	
Heart failure	44 (80%)	400 (400–537.8)	
Hepatic hydrothorax	6 (10.9%)	400 (400–469.2)	
Nephrotic syndrome or dialysis	1 (1.8%)	400 (400–400)	
Other**	4 (7.3%)	548.5 (400–698.2)	

Abbreviations PE = pleural effusion.

*2 melanoma, 2 urologic cancer, 1 metastatic soft-tissue sarcoma, 1 oesophagus carcinoma, 1 hepatocellular carcinoma, 1 kidney carcinoma, 1 colon adenocarcinoma.

**2 pericarditis, 1 amyloidosis, 1 non-specific.

Data are presented as the median (25th–75th percentiles).

^a^*p* < 0.001 BPE vs MPE (Mann–Whitney U-test). ^b^*p* < 0.001 BPE vs Transudate (Mann–Whitney U-test).

Calprotectin levels were significantly higher in the PF from patients with BPE than from samples obtained from patients with MPE (*p* < 0.01) or transudate (*p* < 0.001). The calprotectin concentration also differed between sub-groups of patients with BPE; patients with tuberculous PE had median concentration of 24,000 ng/mL (6,850 to 24,000 ng/mL) while those with parapneumonic PE had a median concentration of 24,000 ng/mL (7,481 to 24,000 ng/mL).

**Figure 1:**
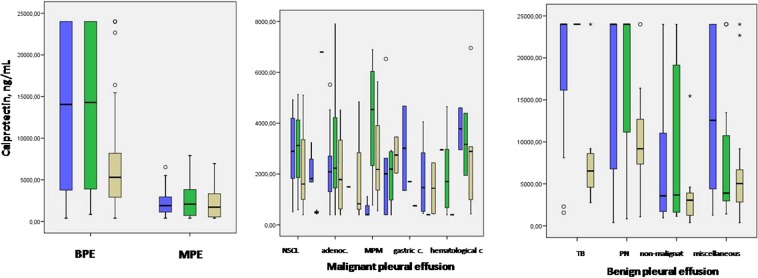
Distribution of concentrations of calprotectin by BPE and MPE (**a**); causes of MPE(**b**) and causes of BPE (**c**). Abbrevations: BPE = bening pleural effusion; MPE = malignant pleural effusion; adenoc = adenocarcinoma; MPM = malignant pleural mesothelioma. c = cancer; TB = tuberculous; PN = paraneumonic.

Patients in the non-MPE group had a median concentration of 3,216 ng/mL (1,718.3 to 11,597.5 ng/mL), whereas the miscellaneous group had a median concentration of 5,202.4 ng/mL (3,142.8 to16,304 ng/mL). We did not find any significant differences in the calprotectin concentrations between groups with different MPE aetiologies. The calprotectin concentrations in PE samples from the three different centres are provided in Supplementary Table S3. Figure 1 shows the distribution of the calprotectin concentrations in pleural fluid by based on the PE cause.

We evaluated the usefulness of calprotectin to differentiate MPE from BPE. ROC curve analysis for a combined total of 360 samples from the three centres showed an AUC value of 0.848 [95% CI, 0.810–0.886]. For a cut-off value of ≤6,233.2 ng/mL, it had a sensitivity of 96% and a specificity of 60%, with negative and positive predictive values and negative and positive likelihood ratios (NLR and PLR) of 96%, 57%, 0.06, and 2.4, respectively. The diagnostic accuracy results for calprotectin levels in PE for predicting MPE, classified by centre and for the entire population are shown in Supplementary Table S4. In the group of patients with MPE with negative cytology results (35), the AUC was 0.855 [95% CI, 0.803–0.907]. For the same cut-off value of ≤ 6,233.2 ng/mL, the sensitivity and specificity were 97% and 60%, respectively.

**Figure 2:**
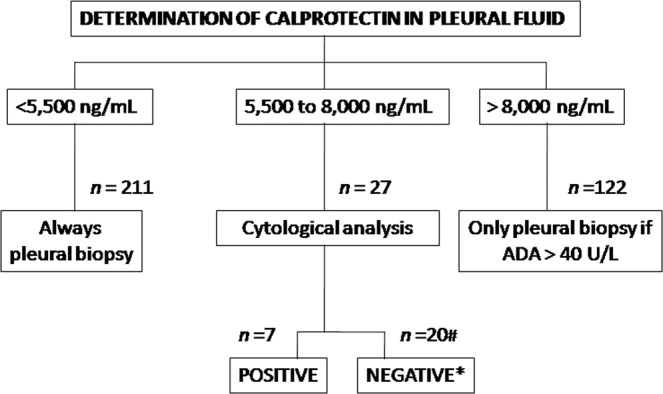
Figure 1. Diagnostic algorithm of pleural effusion including the determination of calprotectin in pleural fluid. Abbreviations ADA: adenosine deaminase; MPE: malignant pleural effusion. *Pleural biopsy when: ♀, radiological size >2/3, previous neoplasia, weight loss, absense of fever, low ADA. ^#^Only one case with MPE: mesothelioma.

The relationship between the malignant origin of PE and other variables, including calprotectin, was also analysed in a univariate analysis (Table 5). These variables were entered into a multivariate analysis for MPE identification and showed that low calprotectin levels in PF had higher discriminatory properties than any other variable [OR 28.76 (*p* < 0.0001); (Table 5)]. Finally, we proposed an algorithm for the diagnostic management of suspected MPE which includes calprotectin as an additional test (Fig. 2).

**Table 5:** Results of the univariate and multivariate logistic regression analysis of the patient demographic characteristics and selected markers.

Characteristics	Univariate analysis OR (95% CI)	P-value	Multivariate analysis OR (95% CI)	p-value
Gender = female	1.49 (0.96–2.30)	0.071	3.31 (1.60–6.83)	0.001
Age 61.5	2.24 (1.406–3.597)	0.001	1.40 (0.69–2.85)	0.344
Smoker	1.21 (0.791–1.86)	0.373	—	—
Previous neoplasia	2.086 (1.26–3.44)	0.004	2.12 (1.01–5.23)	0.045
Dyspnoea	3.00 (1.74–5.16)	0.000	1.46 (0.63–4.43)	0.333
Pain chest	0.577 (0.37–0.90)	0.015	1.25 (0.64–2.46)	0.503
Weight loss	4.55 (2.37–8.75)	0.000	6.70 (2.34–19.13)	0.000
Fever	0.11 (0.04–0.25)	0.000	0.29 (0.09–0.89)	0.032
Cough	0.67 (0.428–1.07)	0.100	1.35 (0.64–2.82)	0.425
PE size 2/3	3.96 (2.27–6.91)	0.000	2.60 (1.05–6.41)	0.037
ADA 27.4 UL	3.73 (2.16–6.45)	0.000	2.71 (1.16–6.51)	0.021
LDH 753 UL	1.06 (0.67–1.68)	0.777	—	—
Protein	0.75 (0.60–0.94)	0.015	1.25 (0.85–1.83)	0.247
pH 7.37	0.88 (0.56–1.40)	0.604	—	—
Glucose	1.00 (1.00–1.01)	0.018	1.00 (0.99–1.00)	0.958
Lymphocytes 73.5%	3.5 (2.12–5.76)	0.000	2.01 (0.59–0.68)	0.261
Neutrophils (%)	0.97 (0.97–0.98)	0.000	1.00 (0.98–1.02)	0.546
Calprotectin ≤ 6. 233, 2 ng/ml	38.29 (15.07–97.25)	0.000	28.76 (9.37–88.28)	0.000

Abbreviations PE = pleural effusion; ADA = adenosine deaminase; LDH = lactate dehydrogenase; OR = odds ratio, CI = confidence interval.

## Discussion

This study confirms the value of calprotectin as a new diagnostic biomarker for PE which might be a useful complement for inclusion in a diagnostic algorithm and would therefore avoid the need for more invasive procedures in some cases. Our results confirmed those obtained in our previous studies^15,16^. In this present study, the determination of calprotectin was adjusted to the workflow of the clinical laboratories at the three participating centres. Calprotectin levels were measured in conventional robotised equipment using a sandwich ELISA kit from Bühlmann Laboratories, which was extensively tested in our laboratory before initiating this present study^18^.

We found that the calprotectin levels in BPE samples were higher than those in the MPE samples, and showed a good accuracy for predicting MPE. The pooled sample ROC curve analysis produced an AUC value of 0.848 and with a selected cut-off, an elevated sensitivity for MPE diagnosis exceeding that of cytological analysis and thus, one which would help to identify patients with a suspected neoplasia. Moreover, calprotectin had a very good NLR, which is especially important when considering new diagnostic biomarkers.

Univariate and multivariate analysis also demonstrated a strong association between low levels of calprotectin and malignancy. Multivariate analysis revealed significant associations between MPE and other variables, however, the contribution of these factors was minimal compared to that of the calprotectin levels. In our study, the patients with a radiological PE size greater than two thirds had higher risk of MPE, which is in line with other evidence that large PEs are usually associated with a higher probability of MPE^19^.

In addition, women had a higher probability of MPE, although this may be because the cases with MPE in female patients were often associated with ovarian or breast cancer. Patients with a previous neoplasia or with symptoms such as weight loss, absence of fever, and low ADA levels in the PF were more often associated with MPE than with BPE in this study, and this agrees with previous reports of associations in patients with MPE^2,3^.

Transudates showed very low calprotectin levels, probably because of the non-inflammatory and/or non-infective condition of these patients. In our study, none of the patients with MPE presented the criteria required for a transudate diagnosis. Nonetheless, several studies have documented cases of MPEs (up to 10%) that are biochemically classified as transudates^19,20^, and so calprotectin should be used after confirming the exudative origin of PE.

Of note, the levels of calprotectin reported by Centre 3were lower than that from the other two centres. One explanation may lie in the fact that fewer patients had inflammatory diseases (tuberculous or parapneumonic conditions) in this centre (52% in Centre 1 and 61% in Centre 2, versus 38% in Centre 3). Another explanation might be that, unlike Centres 1 and 2, Centre 3 did not use anticoagulants during the sample collection procedures. Nonetheless, we did not find any differences between the CI of the ROC curves for the three centres, thus demonstrating the diagnostic accuracy of calprotectin in a routine clinical scenario.

Calprotectin has been studied as a biomarker for several different diseases, and its main current use in the assessment of inflammatory bowel diseases by analysing faeces^21^. However, calprotectin has been studied as a diagnostic marker in urine for the diagnosis and staging of bladder cancer^22^. We were only able to identify one study that used calprotectin as a marker in PE to analyse the efficacy and diagnostic accuracy of a combination of calprotectin and CXCL12^23^. These authors showed a low sensitivity (25.6%) but a specificity of 95.4%, although it is worth noting that, compared to this present study, they included fewer patients and only examined the results from one centre.

In the algorithm for the diagnostic management of suspected MPE we propose here, calprotectin levels could be determined simultaneously alongside other biochemical parameters. Patients with calprotectin levels <5,500 ng/mL would be submitted for a pleural biopsy. Patients with levels between 5,500 ng/mL and 8,000 ng/mL and a positive cytology result would be diagnosed with a malignancy while those who are cytologically negative will receive individualised follow-ups, although a biopsy will be especially recommended for female patients, those with a history of neoplasia, a PE radiological size exceeding two thirds of the chest, low ADA levels, or weight loss in the absence of fever. Finally, patients with calprotectin levels over 8,000 ng/mL would be referred for a biopsy if their ADA levels are also high^4^.

When we applied this algorithm in our settings, there were no false negatives, and only one patient had high PF calprotectin levels (6,888.95 ng/mL) and was subsequently diagnosed with a neoplasm (mesothelioma). However, it is important to consider that this disease is a rare form of cancer. CT has also been reported to have a high specificity for the diagnosis of MPE^24^ and so it is likely that we would have found similar findings if we had also analysed CT.

Thus, based on our findings, we propose that the PF calprotectin concentration should be measured in any patients with suspected MPD, and then, based on the standard clinical, radiological, and biochemical characteristics, cytohistological techniques will be performed. This would improve diagnostic accuracy and allow a new predictive scale to be created in order to improve the indication for more invasive tests. Therefore, future multicentre studies should aim to create, test and potentially validate this algorithm.

One limitation of this study was that it only represented one region in Spain. Thus, these findings must be confirmed in other populations with different ethnic and racial profiles. The development of a technology that could allow the immediate determination of calprotectin alongside the standard biochemical and cytological parameters^25^, especially in a rapid and cost-effective form (i.e. a monotest), would also be of great interest. Finally, each centre could choose to determine its own calprotectin cut-off points in order to overcome potential differences in sample processing.

In summary, measuring calprotectin in patients with PE with an uncertain aetiology would improve diagnostic accuracy and help clinicians in the PE diagnosis decision-making process because it helps to exclude the possibility of MPE in certain cases, thus avoiding invasive procedures.

## Supplementary information

Supplementary information

## References

[1] Thomas JM, Musani AI (2013). Malignant pleural effusions: a review. Clin. Chest. Med..

[2] Roberts, M. E., Neville, E., Berrisford, R. G., Antunes, G. & Ali, N. J. BTS Pleural Disease Guideline Group Management of a malignant pleural effusion: British Thoracic Society Pleural Disease Guideline 2010. *Thorax*. **65**(Suppl2), ii32–ii40 (2010) 10.1136/thx.2010.136994 20696691

[3] Wu SG (2013). Survival of lung adenocarcinoma patients with malignant pleural effusion. Eur. Respir. J..

[4] Villena Garrido V (2014). Recommendations of diagnosis and treatment ofpleural effusion. Update. Arch. Bronconeumol..

[5] Assawasaksakul T, Boonsarngsuk V, Incharoen P (2017). A comparative study of conventional cytology and cell block method in the diagnosis of pleural effusion. J. Thorac. Dis..

[6] Arnold DT (2018). Investigating unilateral pleural effusion: the role of cytology. Eur. Respir. J..

[7] Koegelenberg CF, Diacon AL (2013). Image-guided pleural biopsy. Curr. Opin. Pulm. Med..

[8] Karpathiou G, Stefanou D, Froudarakis ME (2015). Pleural neoplastic pathology. Respir. Med..

[9] Froudarakis ME (2012). Pleural diseases in the molecular era-time for more answers: Introduction. Respiration..

[10] Sriram KB (2011). Diagnostic molecular biomarkers for malignant pleural effusions. Future Oncol..

[11] Tian P (2014). Diagnostic value of survivin for malignant pleural effusion: a clinical study and meta-analysis. Int. J. Clin. Exp. Pathol..

[12] Fafliora E, Hatzoglou C, Gourgloulianis KI, Zarogiannis SG (2016). Systematic review and meta-analysis of vascular endothelial growth factor as a biomarker for malignant pleural effusions. Physiolo Rep..

[13] Ren R (2016). Diagnostic value of fibulin-3 for malignant pleural mesothelioma: a systematic review and meta-analysis. Onco Target..

[14] Yang Y, Liu YL, Shi HZ (2017). Diagnostic accuracy of combination of tumor markers for malignant pleural effusion: an updated meta-analysis. Respiration..

[15] Rodríguez-Piñeiro AM, Blanco-Prieto S, Sánchez-Otero N, Rodríguez-Berrocal FJ, Páez de la Cadena M (2010). On the identification of biomarkers for non-small cell lungcancer in serum and pleural effusion. J. Proteomics..

[16] Sánchez-Otero N (2012). Calprotectin: A novel biomarker for the diagnosis of pleural effusion. Brit J. Cancer..

[17] Light RW, Macgregor MI, Luchsinger PC, Ball WC (1972). Pleural effusions: the diagnostic separation of transudates and exudates. Ann. Intern. Med..

[18] Vázquez-Iglesias L (2017). Evaluation of an automated commercial ELISA method for calprotectin determination in pleural fluid. Clin. Chem. Lab. Med..

[19] Heffner JE (2008). Diagnosis and management of malignant pleural effusions. Respirology..

[20] Ferreiro L (2017). Predictive models of malignant transudative pleural effusions. J. Thorac. Dis..

[21] Daj C, Jiang M, Sun MJ (2017). Fecal calprotectin in monitoring the disease activity in colonic inflammatory bowel disease. Dig. Dis. Sci..

[22] Yaser O, Akcay T, Obek C, Turegun FA (2017). Significance of S100A8, S100A9 and calprotectin levels in bladder cancer. Scand. J. Clin. Lab. Invest..

[23] Luo J (2015). A novel combination of calprotectin and CXCL12 for predicting malignacy in patients with exudative pleural effusion. Medicine..

[24] Hallifax RJ (2015). Role of CT in assessing pleural malignancy prior to thoracoscopy. Thorax..

[25] Wang Y (2015). Development of cancer diagnostics—from biomarkers to clinical tests. Transl. Cancer Res..

