# Supplementary material for: Validation of Calprotectin As a Novel Biomarker For The Diagnosis of Pleural Effusion: a Multicentre Trial
Source: Sci Rep. 2020 Mar 30;10:5679. doi: 10.1038/s41598-020-62388-y (PMC7105479; doi:10.1038/s41598-020-62388-y)
Supplement: Supplementary file 1 — Supplementary information [file 41598_2020_62388_MOESM1_ESM.docx]

**VALIDATION OF CALPROTECTIN AS A NOVEL BIOMARKER FOR THE DIAGNOSIS OF PLEURAL EFFUSION: A MULTICENTRE TRIAL**

Maribel Botana-Rial^^1^, Lorena Vázquez-Iglesias^^2^, Pedro Casado-Rey^3^, María Páez de la Cadena^2^, María Amalia Andrade-Olivié^3^, José Abal-Arca^4^, Laura García-Nimo^5^, Lucía Ferreiro-Fernández^6^, Luis Valdés-Cuadrado^6^, María Esther San-José^7^, Francisco Javier Rodríguez-Berrocal^2^, and Alberto Fernández-Villar*^1^

^1^Pulmonary Department, Hospital Álvaro Cunqueiro, EOXI Vigo, PneumoVigoI+I Research Group, Health Research Institute Galicia Sur (IIS Galicia Sur), Spain.

^2^Department of Biochemistry, Genetics and Immunology, Faculty of Biology, University of Vigo, Spain.

^3^Clinical Chemistry Department, Hospital Álvaro Cunqueiro, EOXI Vigo, Spain.

^4^Pulmonary Department, University Hospital Complex in Ourense, EOXI Ourense, Spain.

^5^Clinical Chemistry Department, University Hospital Complex in Ourense, EOXI Ourense, Spain.

^6^Pulmonary Department, University Hospital Complex in Santiago, EOXI Santiago, Spain, Health Research Institute Santiago (IDIS).

^7^Clinical Chemistry Department, University Hospital Complex in Santiago, EOXI Santiago,Spain.

*Correspondence: Alberto Fernández-Villar.

alberto.fernandez.villar@sergas.es

Pulmonary Department, Hospital Álvaro Cunqueiro, Planta −1, Office10, Estrada

Clara Campoamor, nº 341,36312 Vigo (Pontevedra),Spain.

^These authors have contributed equally to this work

**Supplementary Table S1. Demographic characteristics and symptoms of the study population classified by participating centres.**

|  | **BPE** | | | **MPE** | | | **TRANSUDATE** | | |
| --- | --- | --- | --- | --- | --- | --- | --- | --- | --- |
| **Variable** | **Centre 1** | **Centre 2** | **Centre 3** | **Centre 1** | **Centre 2** | **Centre 3** | **Centre 1** | **Centre 2** | **Centre 3** |
| **Gender**(male/female) | 75/44 | 38/16 | 32/18 | 30/25 | 27/17 | 19/18 | 21/8 | 9/3 | 11/3 |
| **Age** (years)^b^ | 64  (50-75) | 64.5  (50-78.5) | 69  (53-80.5) | 72.5  (61-73) | 71  (60.25-81.5) | 74.5  (63.2-81) | 77  (68-85) | 76.5  (67-82.5) | 81  (68.5-87.5) |
| **Tobacco** | 61 (51.3%) | 27 (50%) | 20 (40%) | 32 (57.1%) | 25 (56.8%) | 17 (45.9%) | 13 (44.8) | 8 (66.7%) | 6 (42.9%) |
| **Cancer** | 27 (22.7%) | 3 (5.6%) | 9 (18%) | 16 (28.6%) | 9 (18%) | 17 (45.9%) | 7 (24.1%) | 4 (33.3%) | 2 (14.3%) |
| **Dyspnoea** | 67 (57.8%) | 37 (68.5%) | 37 (75.5%) | 44 (80%) | 41 (93.2%) | 29 (80.6%) | 22 (75.9%) | 12 (100%) | 14 (100%) |
| **Pain chest** | 55 (47.8%) | 31 (57.4%) | 17 (34.7%) | 20 (36.4%) | 16 (36.4% | 10 (27.8%) | 2 (6.9%) | 4 (33.3%) | 3 (21.4%) |
| **Weight loss** | 9 (7.8%) | 3 (5.6%) | 3 (6.1%) | 13 (23.6%) | 18 (40.9%) | 3 (8.3%) | - | 1 (8.3%) | 1 (7.1%) |
| **Fever** | 41 (35.7%) | 26 (48.1%) | 5 (10.2%) | 2 (3.6%) | 5 (11.4%) | - | 3 (10.3%) | 1 (8.3%) | - |
| **Cough** | 46 (39.7%) | 19 (35.2%) | 17 (34.7%) | 21 (38.2%) | 10 (22.7%) | 8 (22.2%) | 9 (31%) | 4 (33.3%) | 1 (7.1%) |
| **Radiological sizeb** | 9 (7.6%) | 4 (7.4%) | 11 (22%) | 14 (25%) | 9 (20.5%) | 21 (58.3%) | 1 (3.4%) | 3 (25%) | 3 (21.4%) |

Abbreviations ^a^BPE=Benign pleural effusion, MPE=malign pleural effusion

Date are presented as absolute frecuencies and percent

^a^Date are presented as the median (25th–75th percentiles)

^b^ Pleural effusion size in the chest radiographs: PE occupying more than two thirds of the chest’ when the PE produced opacification of the entire hemithorax or when the fluid reached the arch of the aorta

**Supplementary Table S2. Biochemical characteristics in pleural fluid, classified by participating centres.**

Abbreviations BPE=Benign pleural effusion, MPE=malign pleural effusion; ADA= adenosine deaminase, LDH=lactate dehydrogenase.

|  | **BPE** | | | **MPE** | | | **TRANSUDATE** | | |
| --- | --- | --- | --- | --- | --- | --- | --- | --- | --- |
| **Variables** | **Centre 1** | **Centre 2** | **Centre 3** | **Centre 1** | **Centre 2** | **Centre 3** | **Centre 1** | **Centre 2** | **Centre 3** |
| **ADA (U/L)** | 27  (20.8-49.6) | 22  (12-33) | 20.2  (15.4-26.1) | 20  (14-25.4) | 21  (15.5-25.5) | 18.5  (14.5-22.7) | 14.8  (11.8-20.7) | 15  (12-17) | 11..1  (9.7-12.7) |
| **LDH (U/L)** | 544.5  (328.7-1177.7) | 516.5  (365.7-1063.5) | 375  (235.5-684.7) | 612.5  (356.2-1007.2) | 597  (356.7-925) | 482  (204-844) | 160  (148-210.5) | 199  (152.7-2386.2) | 162.5  (110.5-213.7) |
| **Protein(g/dL)** | 4.61  (4.2-5.1) | 4.5  (3.4-5) | 3.5  (3-4) | 4.3  (3.7-4.8) | 4.5  (3.7-5) | 3.2  (2.8-3.8) | 2.84  (2-3.4) | 2.3  (2-3.3) | 2.2  (1.6-2.6) |
| **pH** | 7.4  (7.3-7.4) | 7.4  (7.3-7.5) | 7.4  (7.4-7.5) | 7.4  (7.3-7.4) | 7.4  (7.3-7.5) | 7.4  (7.3-7.5) | 7.5  (7.4-7.5) | 7.5  (7.4-7.5) | 7.5  (7.4-.5) |
| **Glucose (mg/dL)** | 96  (72-122.5) | 99  (80.2-121.7) | 83.5  (66.7-99.7) | 107  (90-148) | 109  (92.2-123) | 75.5  (64.2-103.2) | 115  (104-133.5) | 126  (109.2-166.5) | 87.5  (77.5-95.7) |
| **Lymphocytes (%)** | 80  (35-95) | 33.5  (10.7-70) | 75.5  (55.7-90.5) | 90  (82-97) | 51.5  (28-73.2) | 88  (66.2-97.7) | 95  (88.5-98.7) | 81  (34-91) | 88  (70-97) |
| **Neutrophils (%)** | 16.5  (3-56.2) | 49  (14.7-76.5) | 24.5  (9.5-44.2) | 5.5  (1-15.7) | 13  (6-24) | 12  (8.2-33.7) | 5  (1-15.2) | 7  (3.5-38) | 13  (3.7-31.2) |

Date are presented as the median (25th–75th percentiles)

**Supplementary Table S3. Calprotectin concentrations in pleural fluid (ng ml^-1^) by**

**participating centres**

| **Causes of pleural effusion** | **Centre 1** | **Centre 2** | **Centre 3** |
| --- | --- | --- | --- |
| **Benign pleural effusion** | 14060  (3580-24000) | 14291  (3899.7-24000) | 5302  (2874.7-8439) |
| Tuberculous PE | 24000  (10157.9-24000) | 24000  (24000-24000) | 6534.2  (4211.8-8902) |
| Parapneumonic PE | 24000  (6339.2-24000) | 24000  (10976-24000) | 9176  (7322-13143.7) |
| Non-malignant PE | 3580  (1718.3-11597.5) | 3674.4  (1376.9-21570) | 3056.5  (828.5-4267.1) |
| Miscellaneous P^a^ | 12565  (4387-24000) | 3902.9  (2537.6-12113.3) | 5032  (2530-5817.2) |
| **Malignant pleural effusion** | 1902.5  (1118-2952.75) | 2077  (683.5-3867.25) | 1720  (533.52-3338.33) |
| Non small cell lung cancer | 2895  (1501.9-4373) | 3125  (597-5127) | 1607  (400-5098.5) |
| Adenocarcinoma | 2084.5  (1231.9-2711) | 2231.7  (1409-4253) | 1782  (598.1-3336.4) |
| Small cell lung cancer | 1815  (1684-2916.2) | 487  (424-573.9) | 6797 |
| Ovarian cancer | 2006  (400-3597.7) | 2190  (689-2906.9) | 2748  (2039-3458) |
| Gastric cancer | 3011.4  (1351.8-4671) | 1701.6 | 751  (732.2-769.9) |
| Breast cancer | 1466  (510.1-3141.5) | 400 | 1441.6  (442.3-2441) |
| Unknown origin | 1495 | - | 826.3  (400-4849) |
| Hematologic cancers | 2953 | 1704  (539-3377.2) | 400  (400-400) |
| Mesothelioma | 400  (400-938.5) | 4540.4  (1554.7-6463.4) | 2182.5  (552.4-5628.3) |
| Others* | 3777.5  (2952-4603) | 3170  (1946-4394) | 2889.5  (708.2-5015.5) |
| **Transudate** | 400  (400-526) | 400  (400-417.8) | 420.4  (400-741.6) |
| Heart failure | 400  (400-480.5) | 400  (400-423.8) | 547.3  (400-785.1) |
| Hepatic hydrothorax | 400  (400-607.8) | 400 | 400 |
| Nephrotic syndrome or dialysis | - | - | 400 |
| Others** | 623.3  (548.5-698.2) | - | 400 |

Abbreviation PE = Pleural effusion. Date are presented as the median (25th–75th percentiles)

*2 melanoma, 2 urologic cancer, 1 metastatic soft-tissue sarcoma, 1 oesophagus carcinoma, 1 hepatocellular carcinoma, 1 kidney carcinoma, 1 colon adenocarcinoma

**2 pericarditis, 1 amyloidosis, 1 non-specific.

|  | **Sensitivity** | **Specificity** | **PPV** | **NPV** |  | **PLR** | **NLR** | **AUC** |
| --- | --- | --- | --- | --- | --- | --- | --- | --- |
| **Global** | 96.6  (91.3-98.3) | 60  (53.6-66.1) | 57.6  (51-64) | 96.5  (92.1-98.5) |  | 2.40  (2.04-2.83) | 0.06  (0.03-0.15) | 0.848  (0.81.-0.886) |
| **Centre 1** | 98.2  (9.6-99.7) | 63.9  (54.9-71.9) | 56.1  (46.3-65.5) | 98.7  (93-99.8) |  | 2.72  (2.13-3.46) | 0.03  (0.00-0.20) | 0.859  (0.806-0.911) |
| **Centre 2** | 94.6  (82.3-98.5) | 68.9  (56.4-79.1) | 64.8  (51.5-76.2) | 95.5  (84.9-98.7) |  | 3.04  (2.07-4.45) | 0.08  (0.02-0.31) | 0.860  (0.789-0.931) |
| **Centre 3** | 96  (82.3-98.5) | 40  (27.6-53.8) | 53.8  (41.9-65.4) | 90.9  (72.2-97.5) |  | 1.58  (1.24-2.00) | 0.14  (0.03-0.54) | 0.800  (0.709-0.891) |

**Supplementary Table S4. Measures of diagnostic accuracy for calprotectin**

Abbreviations PPV=Positive predictive value, NPV=Negative predictive value, NLR=Negative likelihood ratio, PLR=Positive likelihood ratio , AUC= area under curve

Date are presented as per cent and confidence intervals of 95%
